# Color discrimination in fixed saturation level of patients with acute traumatic injury

**DOI:** 10.3389/fneur.2024.1363167

**Published:** 2024-04-10

**Authors:** Leonardo R. Nicolau da Costa, Joyce B. Sousa, Felipe André C. Brito, Yuzo Igarashi, Janildes Maria Silva Gomes, Carlos Augusto Lobão, Marcelo Fernandes Costa, Leticia Miquilini, Givago Silva Souza

**Affiliations:** ^1^Instituto de Ciências Biológicas, Universidade Federal do Pará, Belém, Brazil; ^2^Hospital Metropolitano de Urgência e Emergência, Belém, Brazil; ^3^Centro de Ciências Biológicas e da Saúde, Universidade do Estado do Pará, Belém, Brazil; ^4^Núcleo de Medicina Tropical, Universidade Federal do Pará, Belém, Brazil; ^5^Instituto de Psicologia, Universidade de São Paulo, São Paulo, Brazil; ^6^Núcleo de Teoria e Pesquisa do Comportamento, Universidade Federal do Pará, Belém, Brazil

**Keywords:** traumatic brain injuries, visual system, ventral stream, visual processing, color vision

## Abstract

**Introduction:**

Traumatic brain injury (TBI) is an important public health concern and that may lead to severe neural sequels, such as color vision deficits.

**Methods:**

We evaluated the color vision of 10 TBI patients with normal cognitive function using a color discrimination test in a fixed saturation level. We also analyzed computerized tomography scans to identify the local of the brain damages.

**Results:**

Four TBI patients that had lesions in brain areas of the ventral visual streams, five TBI patients had lesions inferred in brain areas of the dorsal visual stream, and one TBI patient had lesion in the occipital area. All the patients had cognitive and color vision screened and they had characterized the chromatic discrimination at high and low saturation. All participants had no significant cognitive impairment in the moment of the color vision test. Additionally, they had perfect performance for discrimination of chromatic stimulus at high saturation and similar to controls (*n* = 37 age-matched participants). Three of four TBI patients with lesions in the ventral brain and one patient with lesion in the occipital area had impairment of the chromatic discrimination at low saturation. All TBI patients with lesions in the dorsal brain had performance similar or slightly worse than the controls.

**Conclusion:**

Chromatic discrimination at low saturation was associated to visual damage in the ventral region of the brain and is a potential tool for functional evaluation of brain damage in TBI patients.

## Introduction

Traumatic brain injury (TBI) is worldwide public health concern and is the main cause of mortality among different head traumas (1). Survivors of this injury develop permanent neurological sequelae that dramatically affect the quality of life (2). Although cognitive, motor, and sensory deficits are among the main consequences of TBI (3), visual impairments are also a relatively common occurrence (4). TBI can affect visual function through a direct lesion in cortical areas or in pathways that connect the eyes to the brain (5). Visual functions usually affected by TBI include oculomotor dysfunction, visual acuity impairment, and visual field defects (6). Color vision deficits have been reported to be a sequela of TBI (7–9).

**Figure 1:**
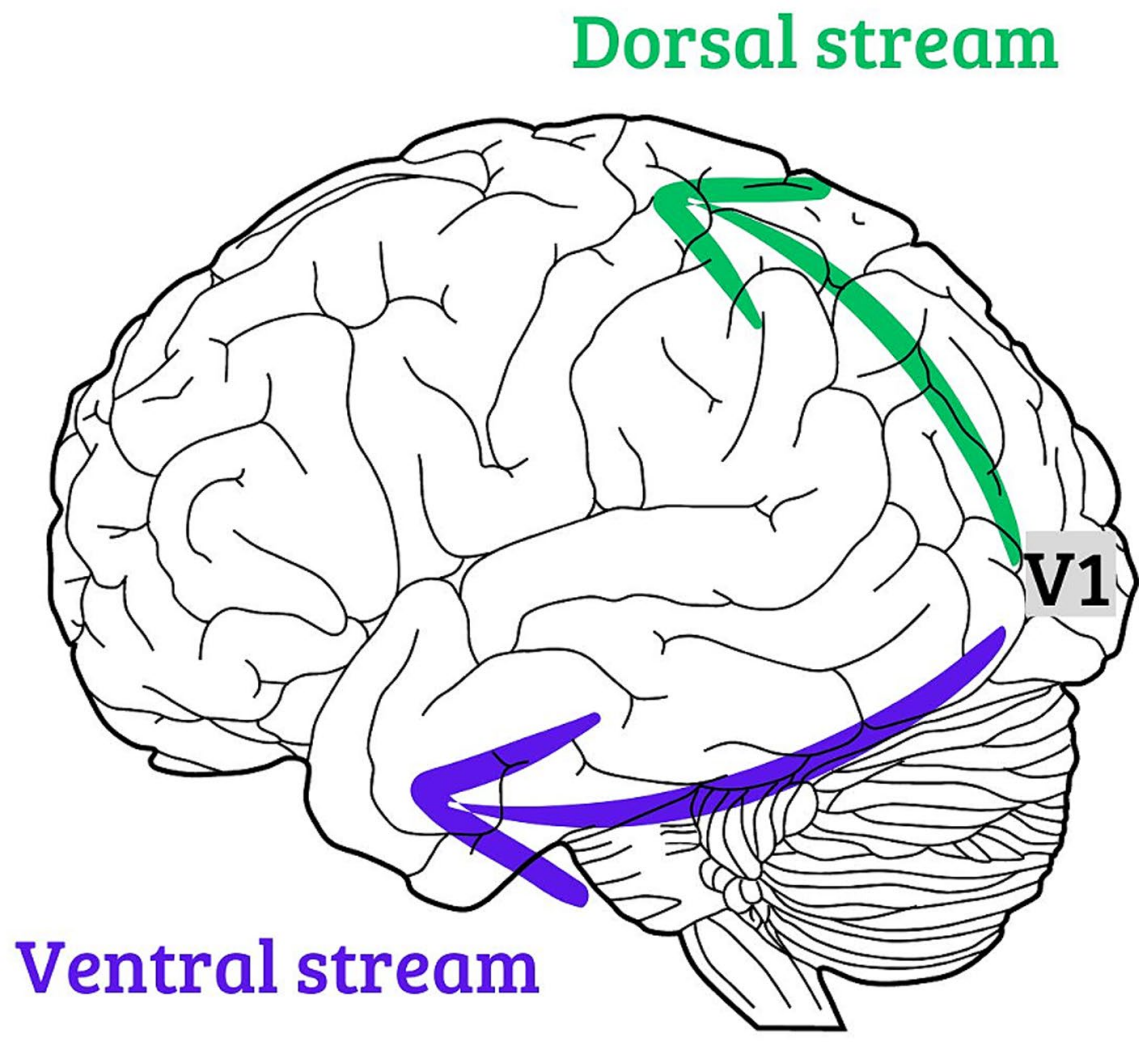
A schematic illustration, depicting location of presumed ventral and dorsal streams of the visual pathway.

There is a hypothesis regarding the functioning of cortical processing of visual information in which the primary visual cortex receives visual input from the retina and the lateral geniculate nucleus, segregating it into two cortical processing pathways (10–12). One destination for the output information from the primary visual cortex would be the lower regions of the temporal lobe, which exhibit biased processing in object properties enabling conscious perception and recognition (13, 14). This pathway is known as the ventral pathway of the visual system. Another destination from the primary visual cortex would be regions located in the posterior parietal lobe, whose processing is biased towards information related to the spatial location of the object and mediates visually guided motor actions (15, 16). This pathway is known as the dorsal pathway of the visual system. Figure 1 shows schematic illustration, depicting location of presumed ventral and dorsal visual streams.

Several studies have documented that color information is mainly processed by the ventral stream of the visual system (17–19), with important contributions from the ventral occipitotemporal cortex and posterior fusiform gyrus (9, 20, 21). In the cortex, color signals are processed along the ventral pathway from V1–V2 to V4, and Inferior Temporal (TE) region (22, 23). Within each of these areas, studies have shown clustering of color-selective regions (24). However, some investigations have shown that color information plays an important role in a cortical region of the dorsal stream of the visual system (19) and that both visual streams interact and influence one another (10).

Studies have reported that damage to localized regions of the cerebral cortex can lead to cerebral achromatopsia, without significant impairment to other visual aspects (25, 26). Total or partial achromatopsia have been reported following ventral visual system disturbances (27). Patients with TBI experience direct damage to several brain areas and are potentially at risk for developing some degree of cerebral achromatopsia.

Many visual tests have been used to identify cerebral dyschromatopsia (27) such as color naming test, Ishihara plates, and Farnsworth-Munsell 100-hue test (26, 28–30). Shin et al. (31) and Igarashi et al. (32) reported how the use of chromatic discrimination in fixed saturation stimuli can be an alternative for rapid screening of chromatic perceptual losses. In this approach, mosaic arrangements are shown with a target and a background that can only be discriminated based on the chromatic saturation difference between them. Our hypothesis is that patients with TBI located in areas associated with the ventral visual pathway would make a greater number of target identification errors compared to patients with injuries located in cortical areas associated with the dorsal visual pathway. The present investigation reports a case series of chromatic discrimination at fixed saturation levels in 10 patients in the acute stage of TBI.

## Methods

### Subjects

The sample consisted of 10 TBI patients who attended the Urgency and Emergency Metropolitan Hospital of Belém, Pará, Brazil, a reference hospital for TBI cases. All patients in the present study had no visual complaints before the trauma they sustained or during the period of visual evaluation, and all had visual acuity of at least 20/40. Patient demographic information and the corresponding causes of TBI are summarized in Table 1. Thirty-seven control age-matched participants were recruited (27.8 years old ±7.4), and all had normal fundus and best-corrected visual acuity to 20/20.

**Table 1:** Demographic features of the TBI patients and information of the TBI history of each patient.

Patient	Age (years old)	Sex	Duration from the trauma to the test (days)	Trauma mechanism
P1	28	M	5	Motor vehicle accident
P2	28	F	10	Motor vehicle accident
P3	45	M	7	Motor vehicle accident
P4	45	M	8	Fall from a height
P5	24	M	40	Motor vehicle accident
P6	30	M	23	Assault
P7	30	M	34	Assault
P8	33	M	8	Fall from a height
P9	28	M	18	Fall from a height
P10	34	M	9	Knife stabbing

M: male; F: female.

All participants provided informed written consent to participate in the present investigation, and all the procedures were approved by the Ethics Committee of the Tropical Medicine Center of the Federal University of Pará (report #2436948).

All TBI patients underwent two screening evaluations using the Mini-Mental State Examination (MMSE) and Ishihara test, and a functional description of their chromatic discrimination using a chromatic discrimination task in fixed-saturation condition. All subjects were binocularly tested. All patients were examined at the time of hospital discharge. The authors also had access to computed tomography (CT) scans of the brain to localize brain area(s) that were potentially damaged.

### MMSE

The cognitive function of the patients was evaluated using the MMSE (33). The examination comprised 10 tasks to evaluate spatiotemporal orientation, registration, attention, calculation, recall, language, reading, repetition, writing, and visuomotor skills. The test score ranged between 0 (worst performance) and 30 (best performance). We considered four cut-off levels to classify the severity of cognitive impairment: score > 24 for no cognitive impairment; score between 19–23 for mild cognitive impairment; score between 10–18 for moderate cognitive impairment; and score ≤ 9 for severe cognitive impairment.

### Ishihara test

The 2016 book version of the Ishihara test with 14 plates was used to screen for problems suggestive of congenital red-green color vision deficiency. Each plate was shown for 3 s under natural daylight. The participants’ task was to indicate the number displayed in the pseudoisochromatic plate. Eight or more errors were considered to represent an altered result (suggestive of dichromacy).

### Chromatic discrimination in fixed saturation level

A chromatic discrimination test followed previous published study (32). The test was programmed in MATLAB environment language (R2017a, Mathworks, Natick, MA, United States). A 15′′ liquid crystal display was used (color resolution, 8 bits per gun; spatial resolution, 1,366 × 768 pixels; temporal resolution, 60 Hz). The display was gamma calibrated using a colorimeter (CS-100A, Konica Minolta, Osaka, Japan).

The test consisted of a pseudoisochromatic stimulus sequence composed of a mosaic of circles randomly distributed across the display (9.7° × 5.4° of visual angle). Luminance noise was applied in the mosaic, in which 6 values of luminance were linearly distributed between 5 and 25 cd/m^2^. A set of circles with chromaticity different from the mosaic field (reference chromaticity: CIE 1976, u’ = 0.219; v’ = 0.48) composing a squared target (1.5° of visual angle).

The participants’ task was to identify where the target was in four alternative positions (up, bottom, left, or right). The test consisted of two stages: pre-test (10 trials); and test (80 trials). In the first 10 presentations (pre-test stage), the chromatic vector of the target chromaticity was 0.07 u’v’ units in the CIE1976 color diagram (high saturated chromaticities) and the 10 chromatic axes between 0 and 342 degrees were chosen randomly to paint the target at each trial. It was avoided that axes on the color confusion lines were chosen. These presentations (2 s duration) were used to evaluate whether the patient understood the commands to be tested. The criterion to follow to the next stage of the test was perfect performance in the high saturated colors stage.

In the second stage of the test, the target chromaticity was shown in 20 chromatic axes (0,18, 36, 54, 72, 90, 108, 126, 144, 162, 180, 198, 216, 234, 252, 270, 288, 306, 324, 342, as shown in Figure 1) in the CIE1976 color diagram, chromatic vector of 0.03 u’v’ units in the CIE1976 color diagram, and four trials of each of the chromatic axes were performed the. Each presentation of the stimulus had 2 s duration and it was interleaved by a dark screen for 1.5 s. The performance of the task was quantified by the number of errors in the identification of the correct target position. In perfect performance, the error value is 0 and, in the worse performance, the error value is 80. Test duration was about 6–7 min.

**Figure 2:**
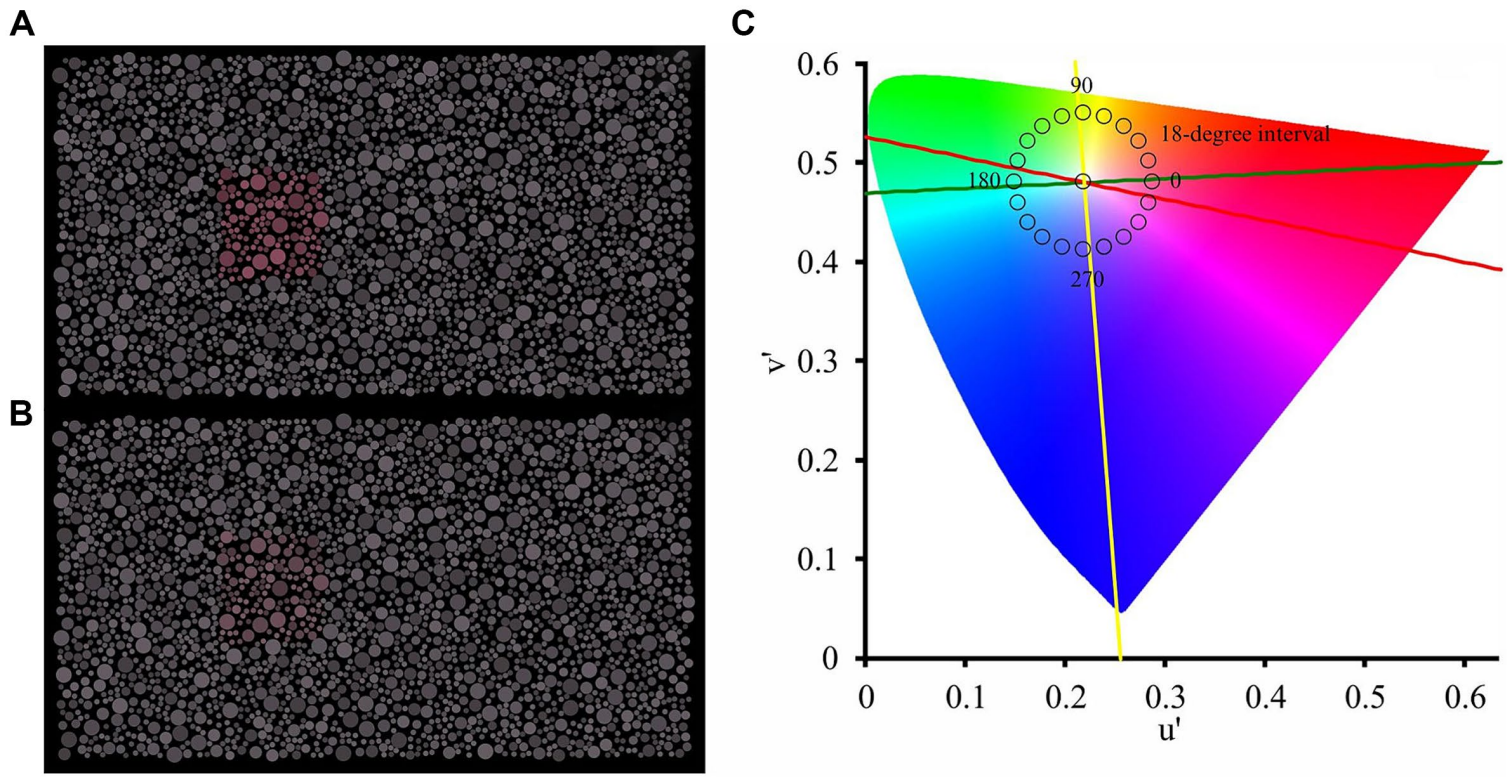
Visual stimulation used in the first stage **(A)** and second stage **(B)** of the chromatic discrimination test. The chromaticity of the target was displayed along 20 chromatic axes spaced at 18-degree intervals in the CIE-1976 color space **(C)**. Yellow line represents the titran confusion line, Red line represents the protan confusion line, and Green line represents the deutan confusion line.

Representative examples of the stimulus in the first and second stages of the test are shown in Figure 2.

### CT scan evaluation

Conventional CT scans of the head were evaluated by an experienced neurosurgeon to identify the location of brain damage.

### Data analysis

To find the cut-off value of the chromatic discrimination performance, we fitted Poisson distribution to the distribution of the number of errors of the control using least mean square method. We considered the 99% percentile in the best fitted binomial function to the data distribution as the cut-off for normal color vision (Normal color vision ≤3 errors, Altered color vision >3 errors). The performance of each TBI patient was compared to this cut-off to be qualified as normal or altered.

## Results

### Cognitive evaluation

Patient scores on the MMSE ranged between 27 and 30, while all controls demonstrated the maximum score (score = 30) in the examination. TBI patients exhibited more difficulty in executing the visuomotor skills task. The MMSE score of each patient and the task in which they exhibited impaired execution is shown in Table 2 leftmost columns.

**Table 2:** Results of the MMSE for each TBI patient and summary of the results obtained from the different evaluations.

Patient	MMSE score*	Color vision test (errors)	Damaged brain region	Task(s) with error
P1	29	5 (altered CV)	Left occipitoparietal (D)	Visuomotor skills
P2	28	2 (normal CV)	Right frontoparietal (D)	Writing and visuomotor skills
P3	28	2 (normal CV)	Right parietal (D)	Writing and visuomotor skills
P4	30	7 (altered CV)	Right temporal (V)	–
P5	29	1 (normal CV)	Left frontotemporal (V)	Calculus
P6	27	45 (altered CV)	Left temporal (V)	Reading, calculus, and writing
P7	28	52 (altered CV)	Right frontotemporal (V)	Writing and visuomotor skills
P8	29	11 (altered CV)	Left occipital	Visuomotor skills
P9	28	4 (altered CV)	Right frontal (D)	Calculation and visuomotor skills
P10	27	1 (normal CV)	Right frontoparietal (D)	Reading, calculation, and writing

* Normal score; D: dorsal streams; V: ventral streams; CV: color vision.

### Ishihara test results

All patients and controls demonstrated perfect performance in the Ishihara test (Table 2).

### Brain imaging results

**Figure 3:**
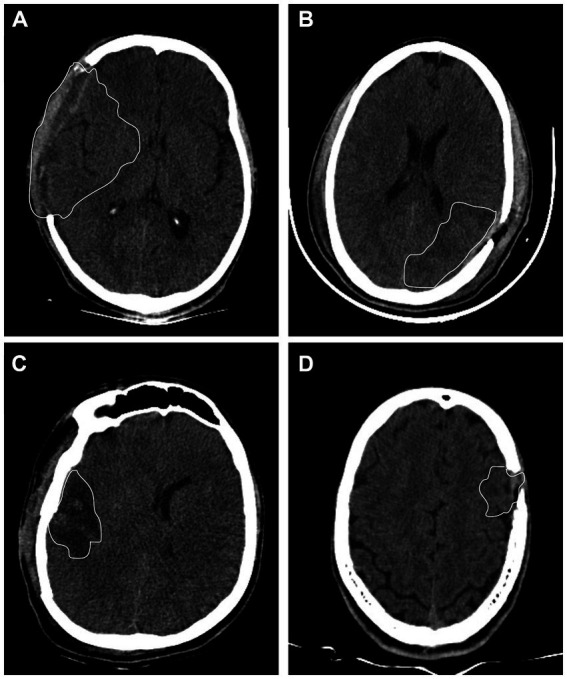
Computerized tomography images from 4 TBI patients. **(A)** Patient P6 had left temporal lesion. **(B)** Patient P7 had frontotemporal lesion. **(C)** Patient P1 had left occipitoparietal lesion. **(D)** Patient P3 had right parietal lesion. White lines represent the visible border of the brain damaged area.

Five patients exhibited lesions in the brain regions belonging to the dorsal visual system (left occipitoparietal region, left parietal region, right frontal region, and frontoparietal region), while four patients exhibited damage in brain areas of the ventral visual system (right frontotemporal region, right temporal region, left frontotemporal region, left temporal region, and right frontotemporal region). One patient had a lesion in the left occipital region, which probably affected both visual streams. The location of brain damage in each patient is shown in Table 2 rightmost columns. CT images of 4 patients are shown in Figure 3.

### Chromatic discrimination in fixed-saturation

All patients and controls showed perfect performance on the first stage of the high-saturation color discrimination test, indicating that they all understood how to perform the task. In the second stage of the test, in which the stimuli with low saturation were presented, the performance of healthy participants ranged from 0 to 4 errors, while the performance of traumatic brain injury patients ranged from 1 to 52, as seen in Table 2.

Patients with lesions in cortical areas related to the dorsal pathway exhibited performances of 1, 2 (2 participants), 4, and 5 errors, while patients with lesions in cortical areas associated with the ventral pathway showed performances of 1, 7, 45, and 52 errors. The sole participant with occipital lobe damage made 11 errors in the test.

**Figure 4:**
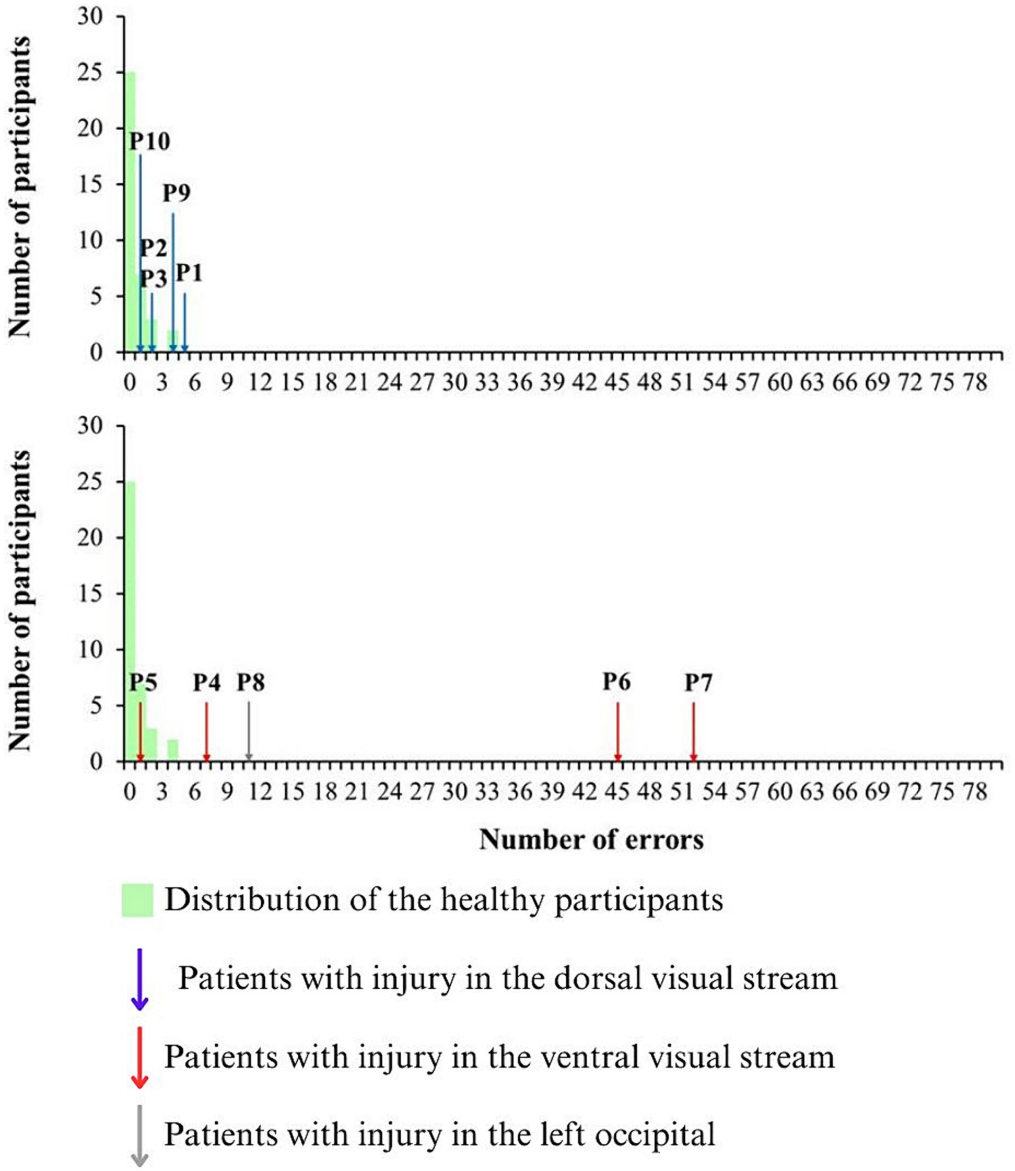
Distribution of the errors in the chromatic discrimination test at low saturated stimulus (green bars). The performance of the patients with inferred dorsal brain lesions are indicated by blue arrows, while the performance of the patients with inferred ventral brain lesions are indicated by red arrows. The performance of the patient with occipital lesion is indicated by gray arrow.

Figure 4 shows the distribution of the control group (green bars) and the indication of the performance from each patient with inferred dorsal stream lesion (blue arrows), inferred ventral stream lesion (red arrows), and in with occipital lesion (gray arrow).

All patients in whom the brain lesion was inferred from the dorsal visual system had errors in the normal range (P2, P3, and P10) or had altered vision with errors just above the cut-off for normal color vision (P1 and P9). Three out of four patients with lesions in the ventral visual stream had errors above the cut-off performance of the controls (P4, P6, and P7) and 1 of them had performance in the range of controls (P5). P6 and P7 had the worst performance among all TBI patients. P6 had a color vision loss especially around the deutan color confusion line and P7 had a diffuse color vision loss. The patient with an occipital lesion (P8) demonstrated more errors than the controls.

## Discussion

Our main finding was that TBI patients with inferred lesions in the ventral visual system stream exhibited poor performance for chromatic discrimination in fixed-saturation than TBI patients with inferred brain lesion in the dorsal visual stream. Because color is mainly processed by the ventral visual stream, its functional impairment could be a non-invasive indicator of a specific lesion of this neural pathway.

We used a computer-controlled portable test that enabled us to screen several chromatic axes relatively quickly (6–7 min). We used a test that displayed pseudoisochromatic stimulus with fixed saturation at the target. In the first stage of our test, we used highly saturated colors to investigate severe impairment of color vision and the patients’ understanding of the psychophysical test what is in agreement with the normal results in Ishihara test. Although this stage of the test is comparable to the Ishihara test, we tested more than red-green vision. This is a first indication of partial color vision loss, since they can discriminate the target in high chromatic saturation. In the second stage of the test, we used a less saturated chromaticity (0.03 u’v’ units in the CIE 1976), which was approximately two times higher than the color discrimination thresholds for adults (34, 35). In this stage, we found that the patients demonstrated errors in different chromatic axes, similar to those observed in other investigations (27).

We consider that our results cannot be explained by a lack of patient comprehension of the test commands. The MMSE scores indicated that all patients had normal cognition (36) in the moment of the test execution, and all of them demonstrated perfect performance on the stage of the test that presented target with high saturated chromaticities (0.07 u’v’ units in the CIE1976 color diagram). We interpreted that errors in the stage of low saturated chromaticities (0.03 u’v’ units in the CIE1976 color diagram) represented an acquired loss of chromatic discrimination.

There is an important debate regarding the existence of a brain area that exclusively processes color information (37–39). The ventral occipitotemporal cortex and posterior fusiform gyrus are brain areas where lesions commonly lead to disturbances of color vision in humans (21, 40). Patients P6 and P7 had the highest error rates in the color discrimination tasks, and their lesions involved the ventral region of the temporal cortex. Patient P8 had the third highest number of errors and had a lesion in the occipital cortex, with probably both streams affected. Patient P4 had the fourth highest number of errors and had a lesion in the right temporal cortex. One case (patient P5) with lesion in the temporal cortex demonstrated good performance in the chromatic discrimination task, and it could suggest that the lesion was possibly anterior to the ventral visual stream. Patients with lesions in the dorsal region of the brain (P1, P2, P3, P9, and P10) performed similarly or slightly worse than the control group.

The number of patients we studied was small and need to be increased to find stronger associations between the location of brain damage and color vision performance. Most similar studies encounter the same limitation; moreover, the study of cerebral achromatopsia is mainly based on case reports or meta-analyses of several different studies (20, 27). Another limitation was that because of the feasibility of application, we implemented the MMSE score as the sole basis to exclude or define cognitive impairment. MMSE is used in clinical setting, however, remains a screening tool rather than a systematic method to diagnose and precisely describe the cognitive impairment, and a much broader battery of tests (including analysis of verbal, executive and memory function, etc.) should be implemented to declare their patients as not cognitively impaired (41). Then, the designation of our patients as having normal cognition should be interpreted with caution.

Our findings suggest that color vision evaluation can be used to assist the diagnosis of functional damage in the ventral visual stream of patients with TBI. The chromatic discrimination test at fixed saturation can be administered quickly and may enable better comprehension of the individual condition of TBI patient’s brain.

## Data availability statement

The raw data supporting the conclusions of this article will be made available by the authors, without undue reservation.

## Ethics statement

The studies involving humans were approved by Ethical Committee of the Federal University of Pará. The studies were conducted in accordance with the local legislation and institutional requirements. The participants provided their written informed consent to participate in this study. Written informed consent was obtained from the individual(s) for the publication of any potentially identifiable images or data included in this article.

## Author contributions

LN: Conceptualization, Formal analysis, Investigation, Methodology, Supervision, Visualization, Writing – original draft, Writing – review & editing. JS: Formal analysis, Investigation, Methodology, Visualization, Writing – review & editing. FB: Formal analysis, Software, Writing – review & editing. YI: Formal analysis, Investigation, Methodology, Writing – review & editing. JG: Formal analysis, Investigation, Writing – review & editing. CL: Conceptualization, Investigation, Methodology, Writing – review & editing. MC: Conceptualization, Formal analysis, Supervision, Writing – original draft, Writing – review & editing. LM: Conceptualization, Funding acquisition, Investigation, Supervision, Writing – original draft, Writing – review & editing. GS: Conceptualization, Data curation, Formal analysis, Funding acquisition, Methodology, Project administration, Resources, Software, Supervision, Validation, Visualization, Writing – original draft, Writing – review & editing.
